# Correlation of perioperative biochemical variables with single adenoma weight in patients with primary hyperparathyroidism

**DOI:** 10.1186/s12893-020-00922-5

**Published:** 2020-11-30

**Authors:** Marios Papadakis, Norbert Weyerbrock, Hubert Zirngibl, Cornelia Dotzenrath

**Affiliations:** 1 Chair of Surgery II, University Witten-Herdecke, Alfred-Herrhausen-Straße 50, 58455 Witten, Germany; 2 Department of Endocrine Surgery, Helios University Clinic Wuppertal, Wuppertal, Germany

**Keywords:** Primary hyperparathyroidism, Adenoma, Weight, Parathormone, Calcium

## Abstract

**Background:**

Single parathyroid adenoma is the main cause of primary hyperparathyroidism (PHPT), with surgery remaining the gold standard for its treatment. The ability to preoperatively predict the parathyroid adenoma size and could facilitate the decision about the extent of surgical exploration. It is reasonable to hypothesize that the perioperative levels of PHPT-related variables (i.e. calcium, parathormone, phosphate) may predict the adenoma weight or/and demonstrate whether the adenoma is successfully removed or not. Aim of this study is to explore the relationship between perioperative biochemical values and adenoma weight. Secondarily, we investigated the relationship between adenoma weight and uni-/bilateral neck exploration.

**Methods:**

Retrospective study of all patients undergone surgery for primary hyperparathyroidism due to single adenoma in a tertiary university hospital in Germany during a 6-year period. Following variables were analyzed: preoperative serum calcium, phosphorus and parathormone, intraoperative parathormone before and after adenoma excision, intraoperative PTH decrease, postoperative serum calcium and parathormone (PTH_postop_—pg/ml), calcium and PTH decrease. Bivariate correlations were calculated by the Spearman's correlation test at the 95% significance level.

**Results:**

A total of 339 patients were included in the study. The median age of the patients was 60 years (range 21–90) and 77% were females. The median adenoma weight was 1 g (range 0.1–11). Adenoma weight correlated strong with maximum adenoma diameter (r = 0.72, p < 0.05), moderate with preoperative parathormone (r = 0.44) and parathormone decrease (r = 0.27), whereas there was no correlation with the intraoperative PTH decrease (r = 0.02). There was also a borderline (moderate to weak) correlation with pre- and postoperative calcium levels (r = 0.21 and r = 0.23 respectively) and a negative borderline correlation with phosphorus (r = − 0.21). Patients who required bilateral neck exploration, had significantly lighter adenomas (median

weight 0.8 g vs 1.1 g, p = 0.005).

**Conclusions:**

We conclude that preoperative PTH levels may only serve as an approximate guide to adenoma weight, as direct preoperative prediction is not possible. Serum calcium levels, PTH and calcium decrease correlate only weak with adenoma weight. Patients who require bilateral neck exploration, have significantly (20–25%) lighter adenomas.

## Background

Single parathyroid adenoma is the main cause of primary hyperparathyroidism (PHPT), responsible for up to 90% of all cases, with surgery remaining the gold standard for its treatment [1]. However, surgical treatment has moved from the traditional bilateral neck exploration to focused surgical techniques, which allow for shorter operation time and lower complication rates.

Parathyroid glands vary in size, number and position. In several cases, initial surgery fails to detect an adenoma, even if imaging investigations strongly suggest its presence. Therefore, the ability to preoperatively predict the adenoma weight, size and location is of great help for the surgeon as it could facilitate the decision about the extent of surgical exploration, indicating larger adenomas in patients with apparently small masses.

It is reasonable to hypothesize that the preoperative levels of PHPT-related variables (i.e. calcium, parathormone, phosphate) may predict the size of a solitary parathyroid adenoma. Moreover, the postoperative levels may also demonstrate whether the adenoma is successfully removed or not. This problem becomes more complex, since the results in the existing literature are very controversial. Multiple authors have shown variable degrees of correlation between serum parathormone (PTH) or/and calcium levels and adenoma weight [2–6], whereas other studies could not confirm these findings [7, 8]. Most studies, though, have a sample size (< 100 patients) that does not allow for conclusive results to be drawn.

Aim of this work is to study the relationship between pre- and postoperative biochemical values and adenoma weight. Secondarily, we investigated the relationship between adenoma weight and uni-/bilateral neck exploration. To the best of our knowledge this is one of the studies with the largest number of parathyroid adenomas studied.

## Methods

### Patient recruitment

We retrospectively reviewed and analyzed the computerized medical records of all patients undergone surgery for primary hyperparathyroidism in a tertiary university hospital in Germany during a 6-year period. The study included patients with single histologically-confirmed parathyroid adenomas. Exclusion criteria included prior thyroid or parathyroid operations, prior neck radiation, patients with causes of hyperparathyoroidism (HPT) others than adenoma, i.e. parathyroid hyperplasia, multiple adenomas, parathyroid carcinoma and multiple endocrine neoplasia (MEN), patients with secondary or tertiary HPT, familial hypocalciuric hypercalcemia (FHH), patients with impaired renal function (serum creatinine > 1.6 mg/dl) and patients unter treatment with calcimimetics (eg. Cinacalcet).

### Procedure

All patients underwent preoperative laboratory assessment, including serum calcium and phosphorus and parathyroid hormone levels (one day before surgery). HPT diagnosis was based on elevated serum calcium and PTH levels. For preoperative parathyroid adenoma localization we routinely performed high-resolution ultrasound and technetium-99 m Sestamibi scan. Neck MRI was reserved for patients with either negative or ambiguous MIBI scan. When the imaging findings were suggestive of single-gland disease, a focused operation was planned and the adenoma was removed with intact capsule and its surrounding fat tissue. All operations were performed or supervised by the same two surgeons to minimize procedural variations.

Intraoperative PTH monitoring was performed in all cases. The measurements were based on peripheral venous blood samples. Four mL of whole blood was collected in a tube with ethylenediaminetetraacetic acid at specific times: (a) preincision baseline, i.e. level prior to skin incision, (b) preoperative baseline, i.e. after dissection and before adenoma's removal and (c) 10 min after excision of the abnormal parathyroid gland. The intraoperative PTH decrease was considered adequate if the Miami criterion was met (drop of 50% or more from the highest, either preoperative baseline or the pre-excision level at 10 min postexcision) and the final value was close to the highest normal levels. In cases of inadequate PTH decrease, an additional measurement was made at 20 min. If the PTH decrease remained inadequate, then further neck exploration was considered and the PTH was measured again after resection of other enlarged parathyroid glands.

Fasting calcium and PTH levels were measured at the first postoperative day. Patients with calcium < 2 mmol/l were given oral substitution and the patients were allowed to leave the hospital once they had reached stable or normal calcium levels. All adenomas were sent to histological examination and the diagnosis was histologically confirmed. The experienced pathologist recorded the weight and dimensions after fat removal. Adenoma weight and diameter were considered the ones reported at the pathologist report.

### Biochemical variables

The following variables were extracted and analyzed (Table 1):

- preoperative (i.e. 1 day before surgery) serum calcium (Ca_preop_, mmol/l), phosphorus (mmol/l), parathormone (PTH_Preop_, pg/ml),
- intraoperative parathormone after adenoma dissection and prior to removal (PTHi_Preop_, pg/ml),
- intraoperative parathormone 10 min − postexcision (PTHi_Postop_, pg/ml),
- intraoperative PTH decrease [DPTHi = (PTHi_Postop_ − PTHi_Preop_) / PTHi_Preop_ × 100%],
- postoperative (i.e. 1st day after surgery) serum calcium (Ca_postop_, mmol/l) and parathormone (PTH_Postop_, pg/ml),
- calcium decrease [DCa = (Ca_Postop_ − Ca_Preop_) / Ca_Preop_ × 100%],
- PTH decrease, from the day before to the 1st postop day [DPTH = (PTH_Postop_ − PTH_Preop_) / PTH_Preop_ × 100%].

**Table 1:** Summary of the biochemical variables analyzed

Definition	Symbol	Description	Unit	Formula
Preoperative calcium	Ca_Preop_	Serum calcium one day before surgery	mmol/l	–
Preoperative Phosphorus	–	Serum phosphorus one day before surgery	mmol/l	–
Preoperative PTH	PTH_Preop_	Serum PTH one day before surgery	pg/ml	–
Intraoperative PTH before resection	PTHi_Preop_	Serum PTH after adenoma dissection and prior to removal	pg/ml	–
Intraoperative PTH after resection	PTHi_Postop_	Serum PTH 10 min postexcision	pg/ml	–
Intraoperative PTH decrease	DPTHi	PTH decrease from just before removal to 10 min postexcision	%	(PTHi_Postop_—PTHi_Preop_) / PTHi_Preop_ × 100%
Postoperative calcium	Ca_Postop_	Serum calcium on the 1st postoperative day	mmol/l	–
Postoperative PTH	PTH_Postop_	Serum PTH on the 1st postoperative day	pg/ml	–
Calcium decrease	DCa	Calcium decrease from the day before surgery to the 1st postop. day	%	(Ca_Postop_—Ca_Preop_) / Ca_Preop_ × 100%
PTH decrease	DPTH	PTH decrease from the day before surgery to the 1st postop. day	%	(PTH_Postop_—PTH_Preop_) / PTH_Preop_ × 100%

### Statistics

**Fig. 1:**
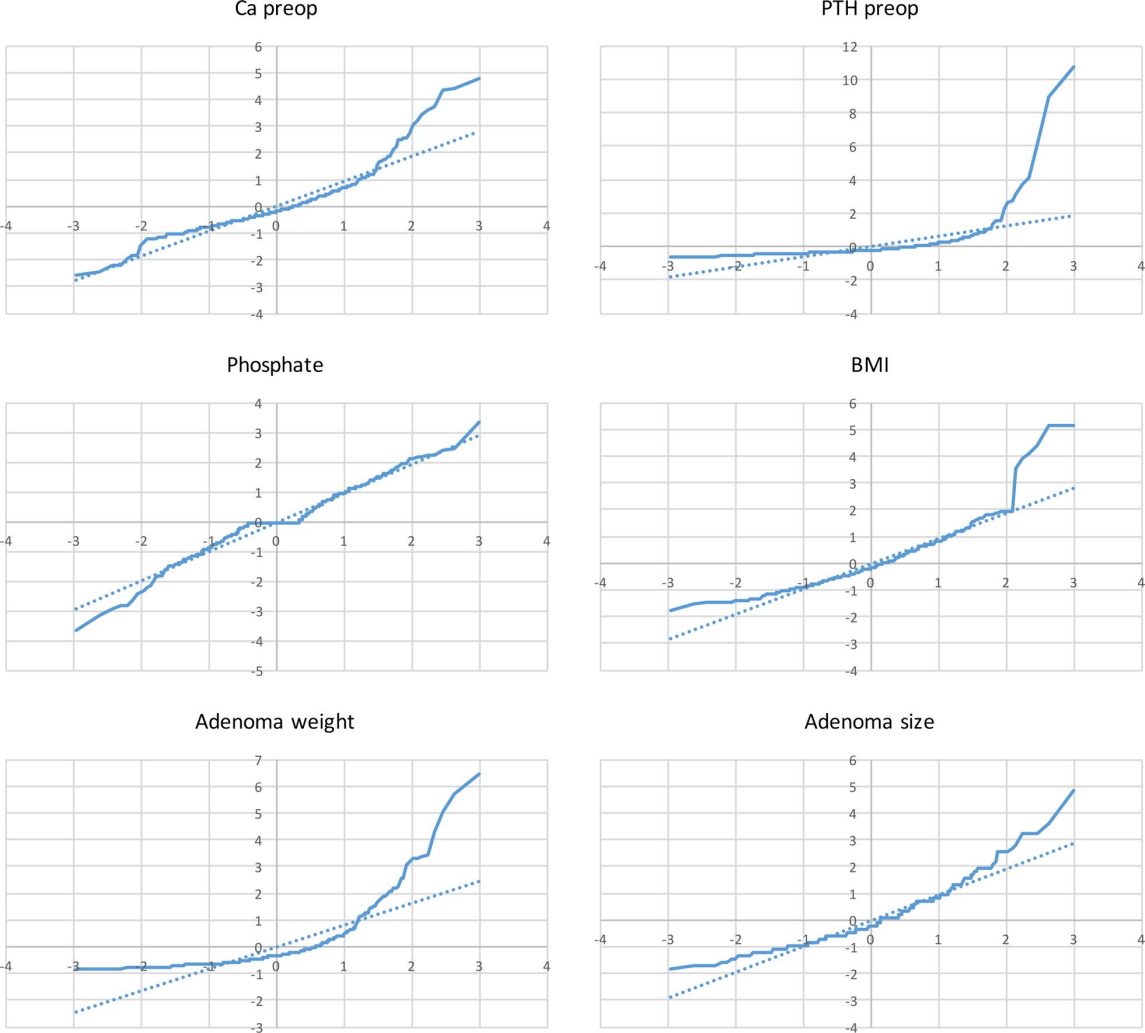
A figure presenting q-q plots providing visual comparison of the sample quantiles to the corresponding theoretical quantiles for four biochemical and two histopathological continuous variables. The deviation of the united line from the dotted (reference) line indicates non-normal distribution. BMI is closer to normal distribution of the parameters examined, followed by phosphate

Normal distribution was determined using histogram plots, Q-Q plots and the Shapiro–Wilk test. Continuous data were not normally distributed and are, therefore, presented in median-range form (Fig. 1). Continuous variables were compared using Mann–Whitney *U* test. Bivariate correlations were calculated by the Spearman's correlation test at the 95% significance level. Correlations were considered strong at r ≥ 0.6, moderate at 0.2 ≤ r < 0.6 and weak at r < 0.2. Data analyses were performed using SPSS 23.

## Results

A total of 339 patients were included in the study and further analyzed. The median age of the patients was 60 years (range 21–90) and 77% were females. The median adenoma weight was 1 g (range 0.1–11). The overall values are presented in Table 2. Adenoma weight correlated strong with maximum adenoma diameter (r = 0.72, p < 0.05), moderate with preoperative parathormone (r = 0.44) and parathormone decrease (r = 0.27), whereas there was no correlation with the intraoperative PTH decrease (r = 0.02). There was also a borderline (moderate to weak) correlation with pre- and postoperative calcium levels (r = 0.21 and r = 0.23 respectively) and a negative borderline correlation with phosphorus (r = − 0.21). All significant correlations are summarized in Table 3.

**Table 2:** Overall biochemical and pathological variables' values in our cohort of 342 patients

Variable	Median (range)
Age (years)	60 (21–90)
BMI (kg/m^2^)	26.5 (16.9–58.1)
Phosphorus (mmol/l)	0.76 (0.26–1.23)
PTH_Preop_ (pg/ml)	122.3 (45–2740)
Ca_Preop_ (mmol/l)	2.83 (2.3–4)
Ca_Postop_ (mmol/l)	2.37 (1.78–3.96)
DCa	16.67%
DPTHi	82%
DPTH	81%
PTH_Postop_ (pg/ml)	23.3 (2.7–305)
Adenoma maximum diameter (cm)	1.8 (0.5–5.8)
Adenoma weight	1 (0.1–11)

Data is not normally distributed and, therefore, is presented in median-range form

**Table 3:** Significant correlations between adenoma weight and study parameters

Variable	r	p-value
Ca_Preop_	0.21	< 0.05
Ca_Postop_	0.23	
PTH_Preop_	0.44	
DPTH	0.27	
Adenoma maximum diameter	0.72	
Phosphorus	− 0.21	
BMI	0.15	

The majority of patients (193, 57%) underwent unilateral neck exploration, whereas the rest 43% (146 patients) was operated bilaterally, for the adenoma to be found and safely excised. The former group consisted of patients with significantly higher adenoma weight (mean 1.7 g, SD 1.86, median 1.1 g, range 0.1–12.2 g vs mean 1.3 g, SD 1.32, median 0.8 g, range 0.1–7.1, p = 0.005), the relationghip being significant but weak (r = − 0.15).

## Discussion

We found only weak correlations between pre- and postoperative biochemical variables and adenoma weight. Although adenoma weight was moderate correlated to preoperative PTH levels, it was not strong enough to be used in the estimation of adenoma weight. Adenoma weight correlates strongly with its maximum diameter, i.e. with its size. This makes possible to use either variable for determining correlation with biochemical markers [8].

The normal parathyroid gland has a mean maximum diameter of 6 mm and a mean weight of 60 mg [8]. In our sample, the mean maximum diameter was 1.8 cm and the mean weight 1000 mg (1 g), values consistent with all western studies. A study from Iran reports much heavier adenomas, attributing the mean weight of 3.8 g to the effect of vitamin D deficiency and low calcium intake on parathyroid glands. The former seems to be the reason why patients in Iran are diagnosed at much younger age compared to the western countries (42 years vs. 60 years in our study) [7].

Several studies could not demonstrate any relationship between serum calcium and PTH and adenoma weight, although these findings may be influenced from patients with hyperplasia, double adenomas and renal hyperparathyroidism, who were not excluded [8]. Kandil et al., in their impressive series of 447 cases, found significant mean weight differences (410 vs. 910 mg) in patients with low and high baseline PTH values (i.e. PTH < or > 150 pg/ml, respectively) [6]. However, they made a comparative study and did not explore correlations in the whole cohort. Strickland et al. report no difference between hypocalcemic and normocalcemic patients with respect to preoperative serum calcium, PTH levels or adenoma weight. They also found higher mean preoperative calcium levels but comparable preoperative PTH and postoperative calcium levels in patients with adenomas > 2 g [9]. However, their method of categorizing a continuous variable may have led to biased parameter estimates and loss of efficiency in predictions [10]. Randhawa et al. dichotomised at several thresholds from 1 to 2.5 g but still failed to identify any adenoma weight predictors.

It is reported that larger parathyroid adenomas secrete PTH at a lower rate than lighter adenomas. This may be explained from the fact that larger adenoma may be filled with inactive zones, i.e. fibrosis, calcification, cystic spaces or hemorrhage into the gland [7, 11]. Another explanation could be that the excess autonomous PTH secretion, triggers negative feedback mechanisms, suppressing PTH secretion by the normal glands. On the other hand, it is not clear if and at which stage of the disease the normal glands cease secretion [8]. Stern et al. found a significant correlation between chief-cell percentage and adenoma weight but there was no correlation with preoperative calcium and PTH levels [3].

Serum PTH levels reflect adenoma and normal parathyroid gland PTH output. It is unclear how much PTH contributes the adenoma but there is evidence suggesting stronger correlation between PTH and adenoma weight in extreme levels of PTH. Therefore, several authors recommend that very high levels of PTH should alarm the surgeon of the possible existence of a too large adenoma [7]. When the PTH level is below 6 pmol/l (57 pg/ml) the adenoma is likely to weigh < 400 mg, whereas PTH levels > 170 pg/ml usually indicate an adenoma weight > 800 mg [2]. The relationship between adenoma weight and calcium is much more complex, as serum calcium values are the result of multiple endocrine calciferol-mediated mechanisms [8].

We found a negative moderate though statistically significant correlation between phosphate and adenoma weight. This is consistent with the studies of Bindlish et al. [5] and Mozes et al. [2] and is not surprising, since hypophosphatemia is observed in about 40% of HPT [2]. Vitamin D deficiency may be associated with heavier parathyroid adenomas, therefore in some departments it is common practice to correct vitamin D deficiency before establishing a HPT diagnosis [3].

According to a large cohort study with > 340 patients, adenoma weight correlates with the percentage decrease in calcium levels from before to after surgery but not with the PTH decrease [3]. Other authors report a significant correlation between adenoma weight and PTH-decrease at 10 min postexcision [12].

In our study, patients who required bilateral neck exploration, had also significantly (20–25%) lighter adenomas. For this reason, bilateral exploration should always be considered in cases of inadequate PTH decrease and adenoma size that can not explain high PTH values, even if imaging studies are strongly suggestive of single-gland disease. Interestingly, additional abnormal glands are present in some patients despite appropriate reduction in IOPTH. Unilateral exploration has been associated with recurrence/persistense rates of 5%, while recurrence rates after bilateral exploration recurrences are < 1% [13].

The controversial literature results can also be associated with other methodological divergences or limitations, except for the above mentioned approach of categorizing a continuous variable. Sadly, the majority of the studies do not report how correlation was tested. Many studies used the Pearson correlation without mentioning if its assumptions were met (normal distribution, homoscedasticity, linearity) [14]. We performed the more conservative Spearman correlation, as our data was not normally distributed [15]. Outliers can also bias the results in small samples, if they are not properly analyzed. Astonishingly, the removal of two heavy adenomas in a series of 44 patients, reduced the correlation coefficient from 0.83 (very strong correlation) to 0.28 (moderate to weak) [11].

Our study has limitations inherent to retrospective studies. Firstly, it is based on medical records, which are captured for clinical purposes. Moreover, the specimens were analyzed from different pathologists during the long study period, with the potential for interobserver bias. Last but not least, the weight of small adenomas may have been overestimated, as their size does not always allow precise removal of the surrounding fat.

## Conclusion

We conclude that preoperative PTH levels may only serve as an approximate guide to adenoma weight, as direct preoperative prediction is not possible. Serum calcium levels, PTH and calcium decrease correlate only weak with adenoma weight. Patients who require bilateral neck exploration, have significantly (20–25%) lighter adenomas.

## Data Availability

The datasets used and/or analysed during the current study are available from the corresponding author on reasonable request.

## Abbreviations

FHH: Familial hypocalciuric hypercalcemia
HPT: Hyperparathyroidism
MEN: Multiple endocrine neoplasia
PHPT: Primary hyperparathyroidism
PTH: Parathormone
